# Supplementary material for: Dual stem cell therapy synergistically improves cardiac function and vascular regeneration following myocardial infarction
Source: Nat Commun. 2019 Jul 16;10:3123. doi: 10.1038/s41467-019-11091-2 (PMC6635499; doi:10.1038/s41467-019-11091-2)
Supplement: Supplementary file 1 — Supplementary info [file 41467_2019_11091_MOESM1_ESM.pdf]

Supplementary Information

Dual stem cell therapy synergistically improves cardiac function and vascular regeneration following myocardial infarction

Park et al.

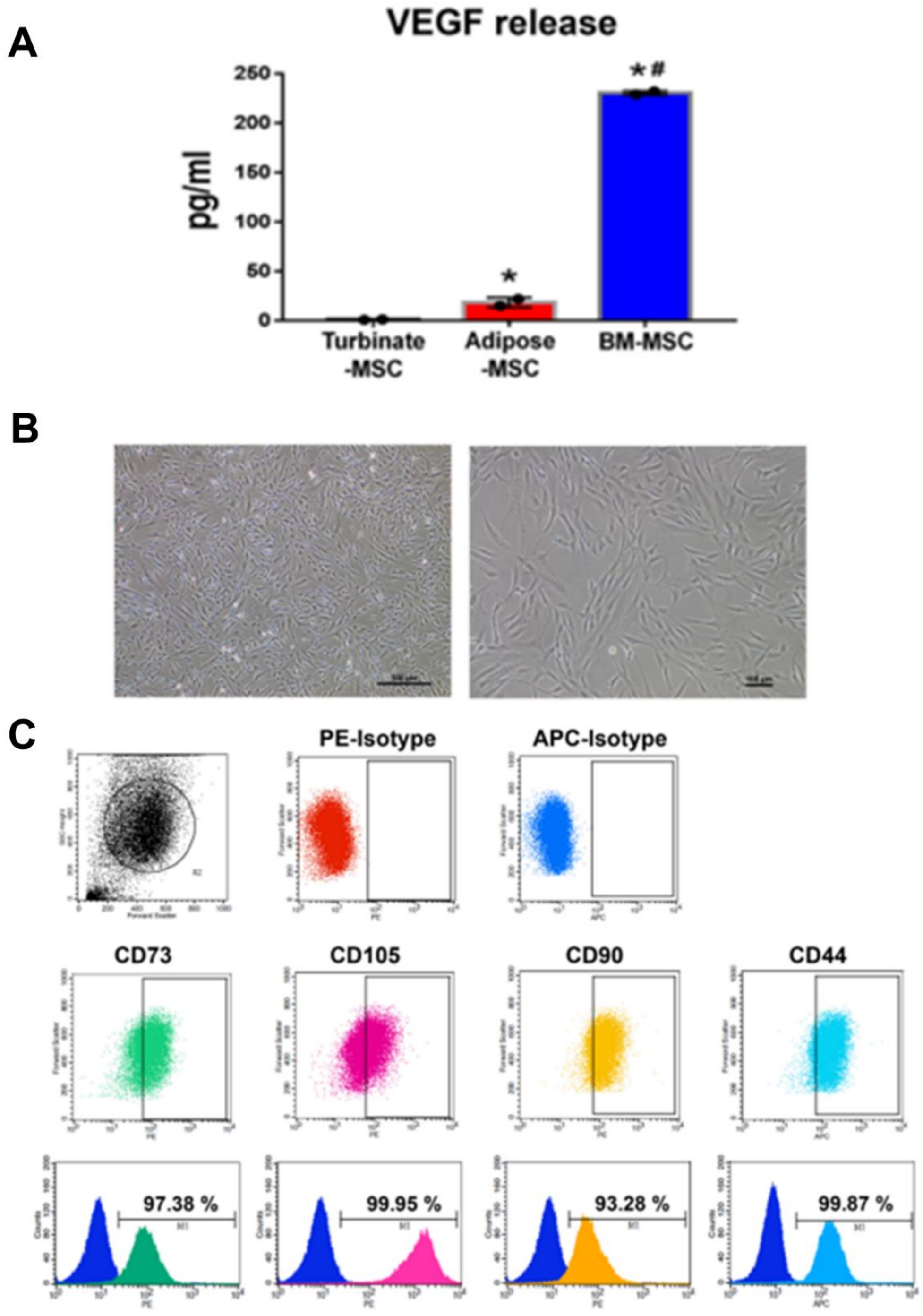

Supplementary Figure 1. Characterization of bone marrow derived hMSCs. (A) Selection of optimal hMSCs based on VEGF secretion measured by ELISA. Data are represented as mean ± SEM. *p < 0.05 compared to human turbinate MSCs, $^{\#}p$ < 0.05 compared to human adipose derived MSCs. n=3 biologically independent samples per group. One way ANOVA was used for statistical analyses. **(B)** Morphology of human bone marrow derived MSCs. **(C)** Flow cytometry analyses show that human bone marrow derived MSC express specific markers for MSCs such as CD73, CD105, CD90 and CD44.

A

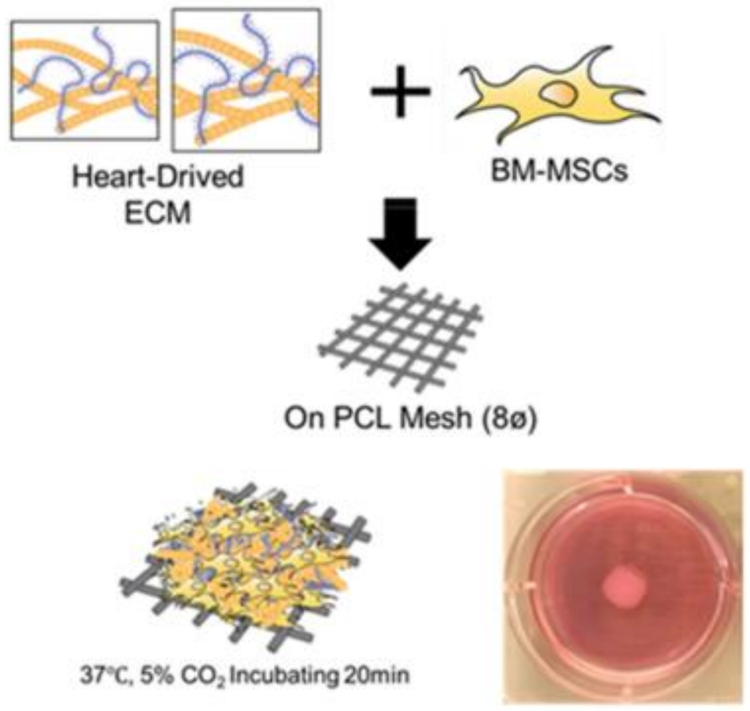

B

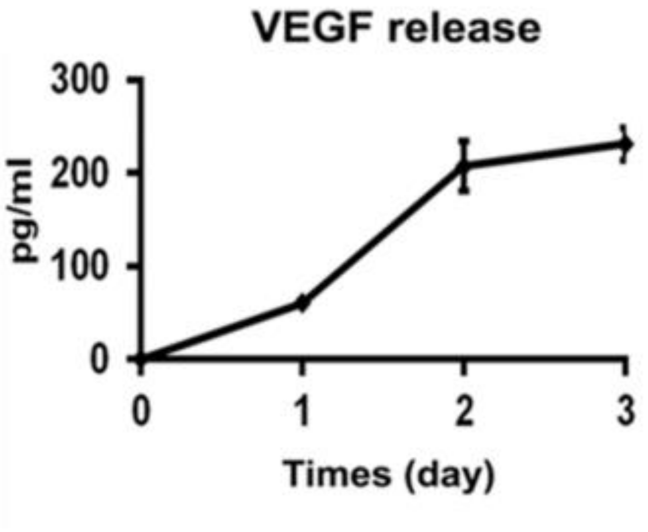

Supplementary Figure 2. (A) Schematic illustration demonstrates the procedures for generating hMSCs-patch using decellularized pig heart tissues derived ECM via 3D printing. **(B)** Measurement of prolonged release of VEGF from hMSCs-patch by using ELISA. Data are represented as mean ± SEM. n=3 biologically independent samples per group.

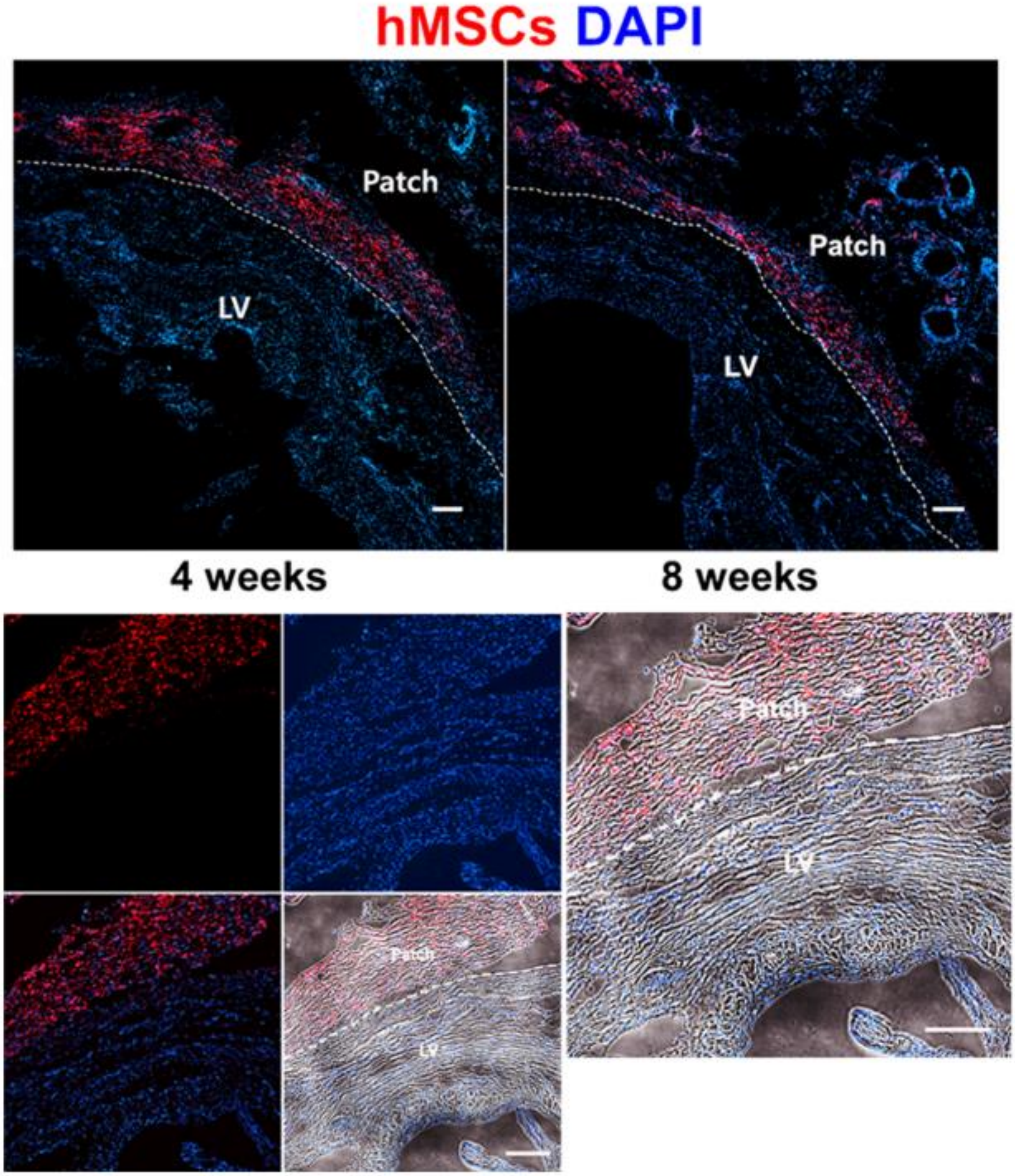

Supplementary Figure 3. hMSCs were located within patch until 8 weeks from implantation. Representative images of DiI labelled human mesenchymal stem cells (hMSCs; red) and DAPI for nucleus (blue) within the patch made by using heart-derived decellularized extracellular matrix (hdECM). Microscopic observations revealed that the majority of DiI positive hMSCs were detectable within the patch. Scale bars: 100µm. n=3 biologically independent samples per group.

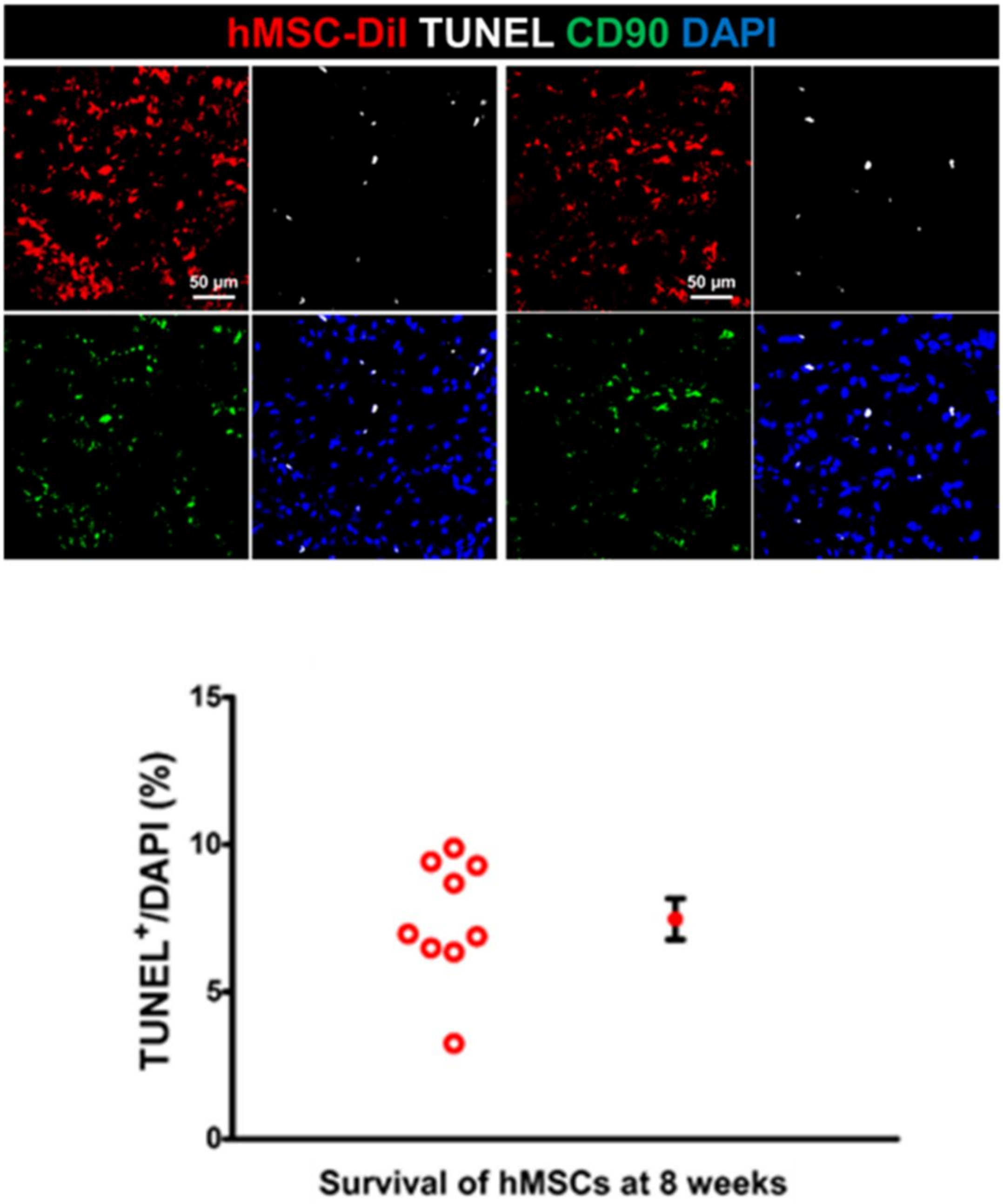

Supplementary Figure 4. Survival of hMSCs within the cardiac patch after 8 weeks from implantation. Representative images of DiI labelled human mesenchymal stem cells (hMSCs; red), TUNEL signal (grey), CD90, a specific protein marker for MCSs (green) and DAPI for nucleus (blue) within the patch made by using heart-derived decellularized extracellular matrix (hdECM). In the TUNNEL assay using the 8 weeks post MI rat heart tissues showed that hMSCs are survived well within the patch until 8 weeks from the implantation. Scale bars: 50µm. Data are represented as mean ± SEM. n=3 biologically independent samples per group.

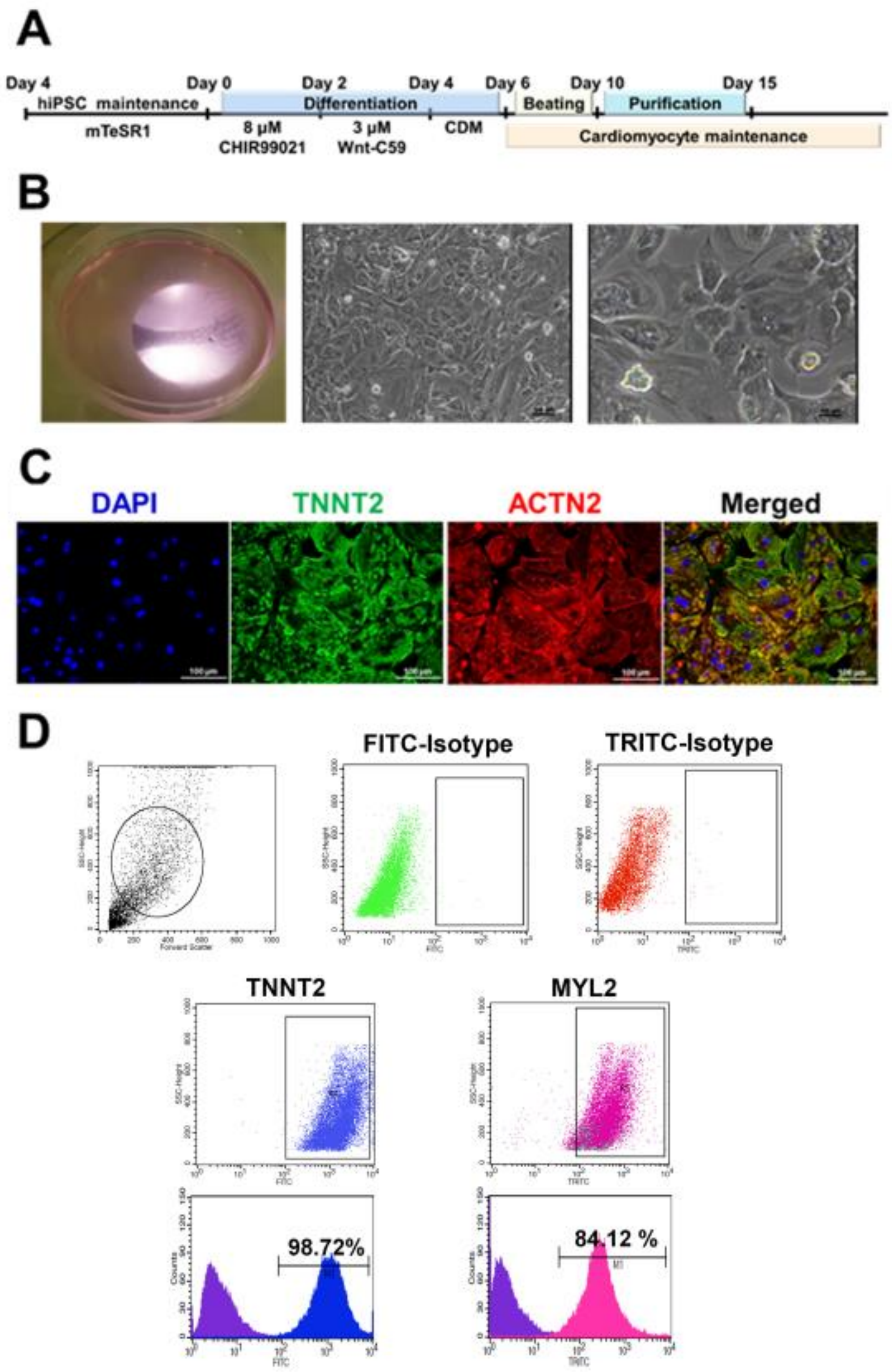

Supplementary figure 5. Generation of cardiomyocytes derived from human induced pluripotent stem cells. (**A)** Schematic of the protocol to differentiate hiPSCs to the cardiac lineage. (**B)** Morphology of differentiated hiPSC-CMs. (**C)** Immunocytochemistry for TNNT2 and ACTN2 on hiPSC-CMs. Scale bars: 100 μm. **(D)** Percent expression of TNNT2 and MYL2 on hiPSC-CMs at differentiation days 15 determined by flow cytometry via CD marker gating; n=3 biologically independent samples per group. . hiPSCs: Human induced pluripotent stem cells; hiPSC-CMs: Cardiomyocytes derived from human induced pluripotent stem cells.

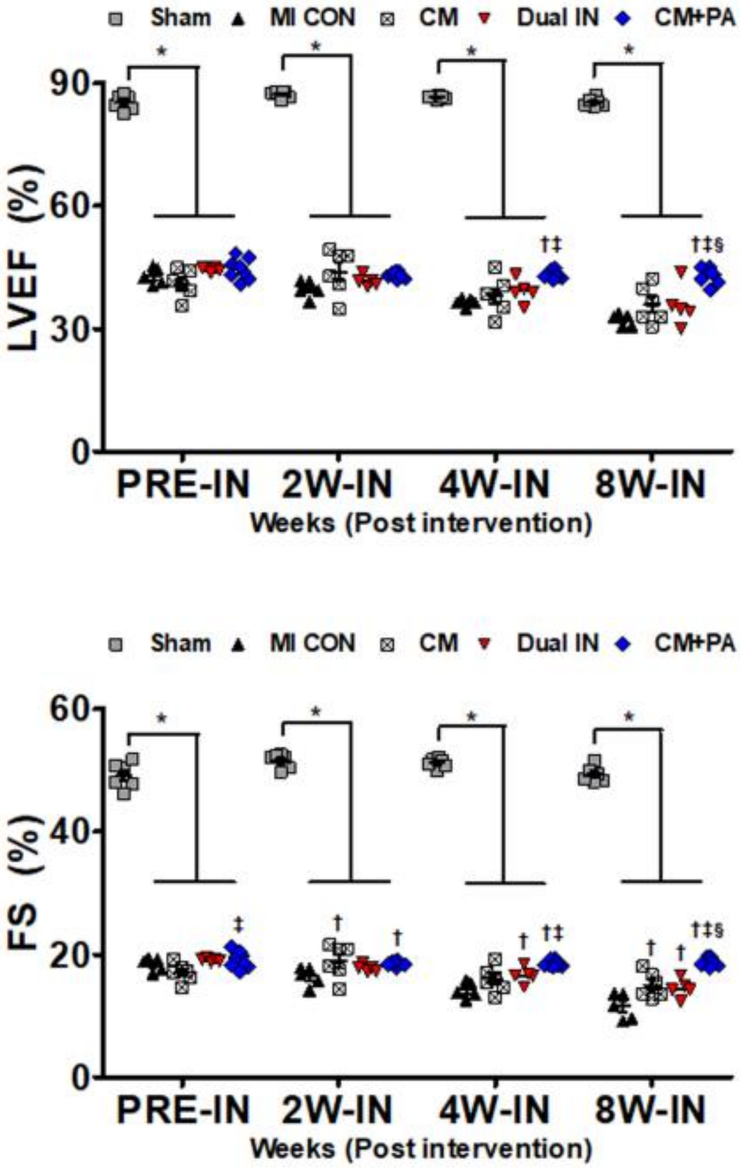

Supplementary Figure 6. Therapeutic effects of dual intramyocardial injection of hiPSC-CMs and hMSCs with hdECM. Cardiac function in rats receiving cell mixture (Dual IN): 1 x 10^6 hiPSC-CMs, and 1 x 10^6 hMSCs together with the hdECM was compared with the dual treatment group with hMSC-patch (CM + PA): 1 x 10^6 hiPSC-CMs injection and the implantation of hMSC loaded patch made by using hdECM. Echocardiography results at 8 weeks demonstrated that the both ejection fraction (EF) and fractional shortening (FS) of Dual IN group was significantly lower than CM + PA group. Data are represented as mean ± SEM. *$p < 0.05$ compared to Sham group, †$p < 0.05$ compared to MI CON group, ‡$p < 0.05$ compared to CM group, §$p < 0.05$ compared to Dual IN group; n=5 animals per group. One way ANOVA was used for statistical analyses. Sham: Sham operation, MI CON: MI control, CM: hiPSC-CM injetion, Dual IN: Injections of both hiPSC-CMs and hMSCs, CM + PA: hiPSC-CMs + hMSC-loaded patch

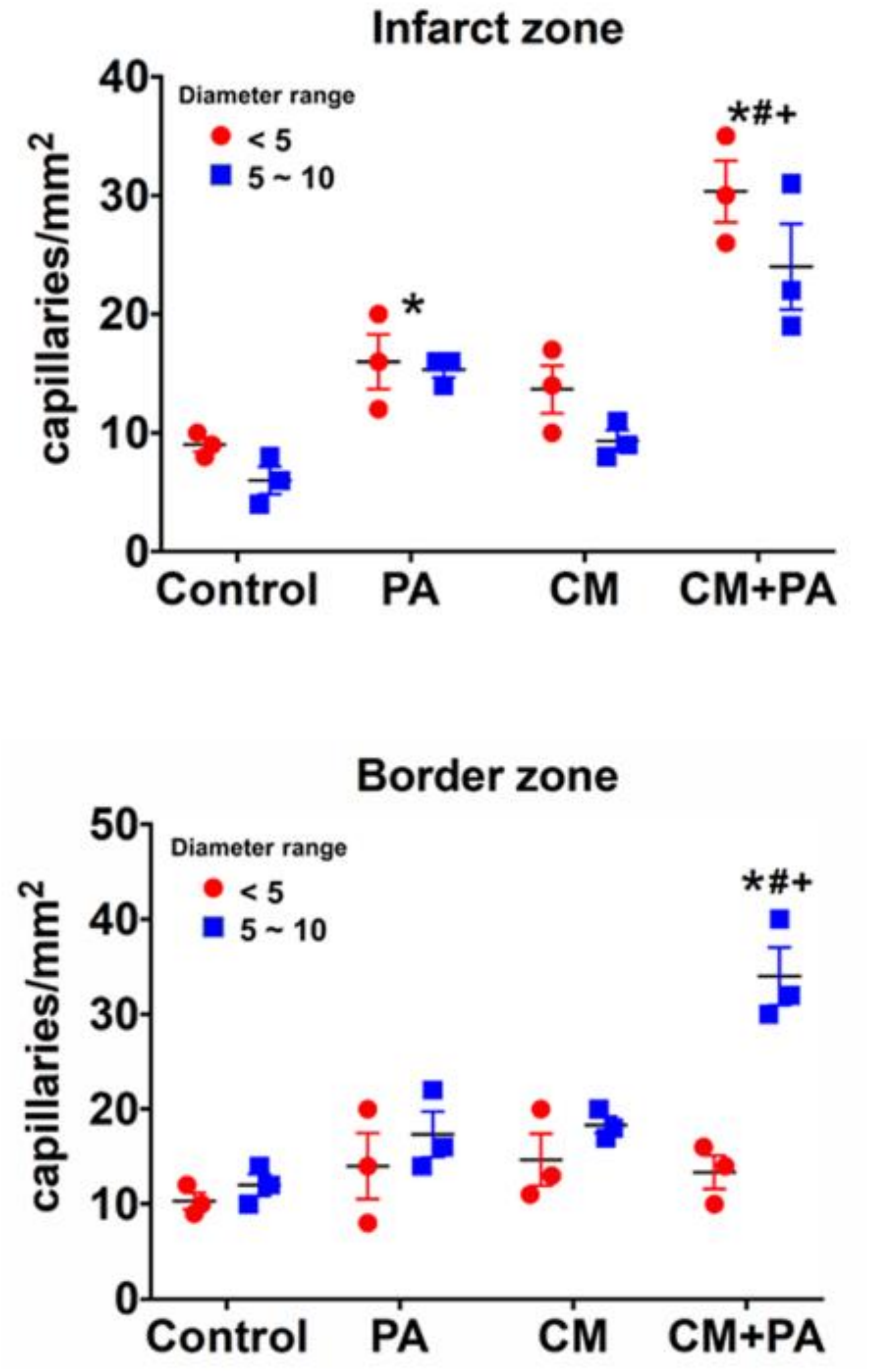

Supplementary Figure 7. Quantification of diameter ranges of the capillaries on the infarct zone, and the border zone at 8 weeks after MI. Data are represented as mean ± SEM. *$p < 0.05$ compared to Control, $^{\#}p < 0.05$ compared to PA group, and $^{+}p < 0.05$ compared to CM group; n=5 biologically independent samples per group. One way ANOVA was used for statistical analyses. Control: MI control, PA: hMSC-loaded patch, CM: hiPSC-CMs, CM + PA: hiPSC-CMs + hMSC-loaded patch

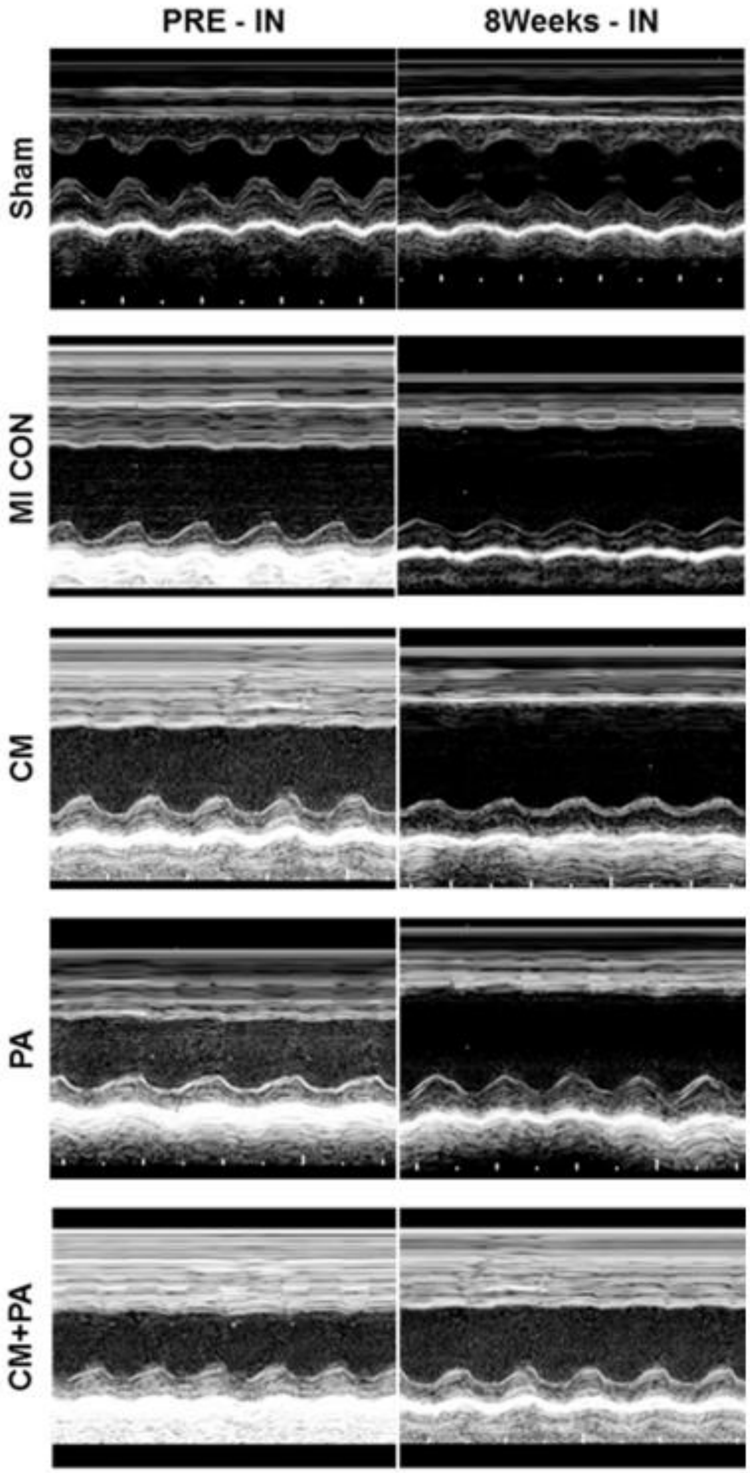

Supplementary figure 8. Representative echo images of all experimental groups at 2 and 8 weeks post interventions.

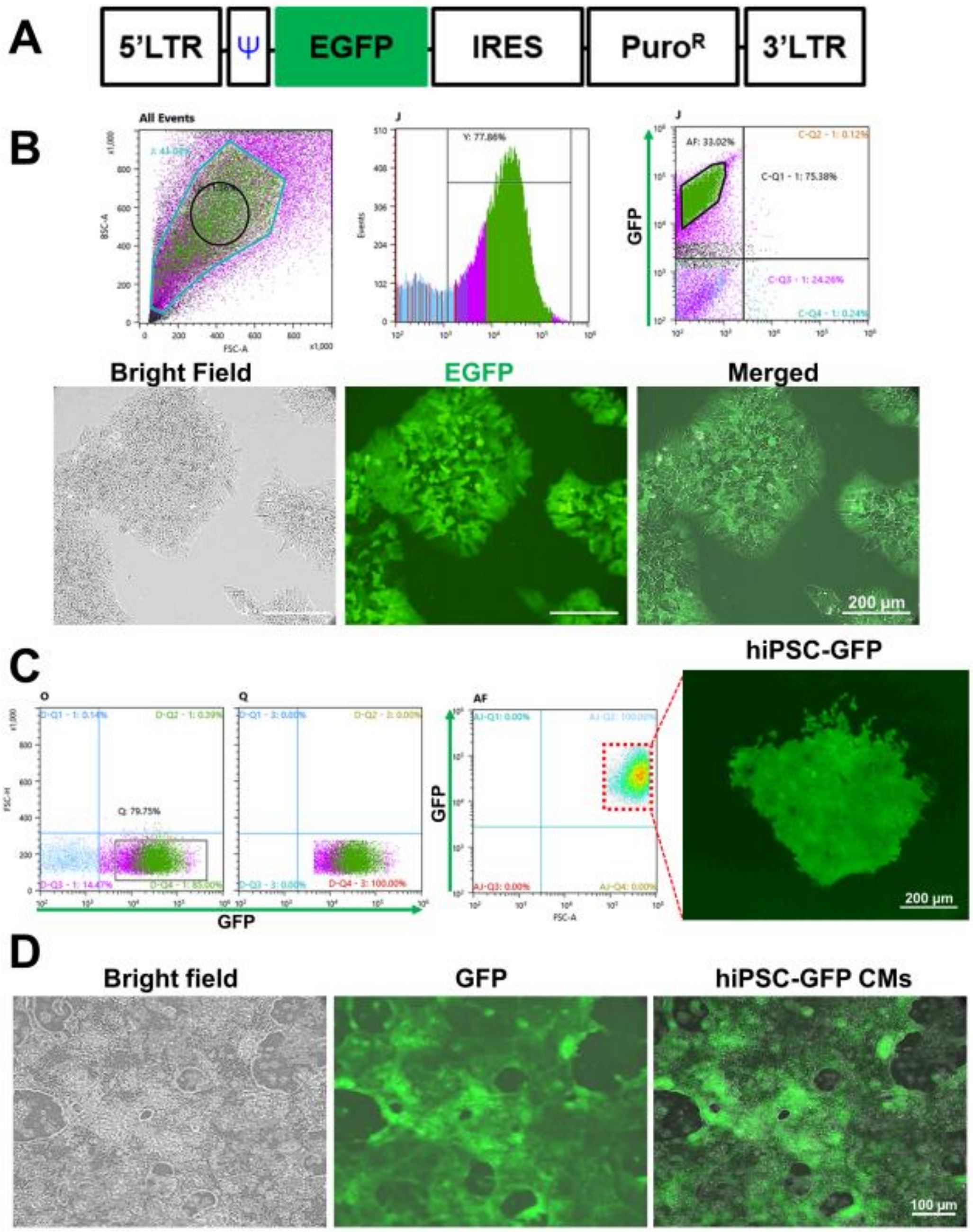

Supplementary figure 9. FACS gating strategy and generation of hiPSC-CM-GFP. (A) Structure of EGFP reporter system. **(B)** Representative images of hiPSC-GFP colony expressing GFP signal as well as FSC and BSC gating followed by GFP for isolation. **(C)** Representative fluorescence-activated cell sorting (FACS) plots showing the percentage of GFP positive hiPSCs (hiPSC-GFP). These hiPSC-GFP were sorted out for subsequent expansion and further differentiation into the cardiomyocytes **(D)** Representative images of hiPSC-CMs-GFP derived from hiPSC-GFP.

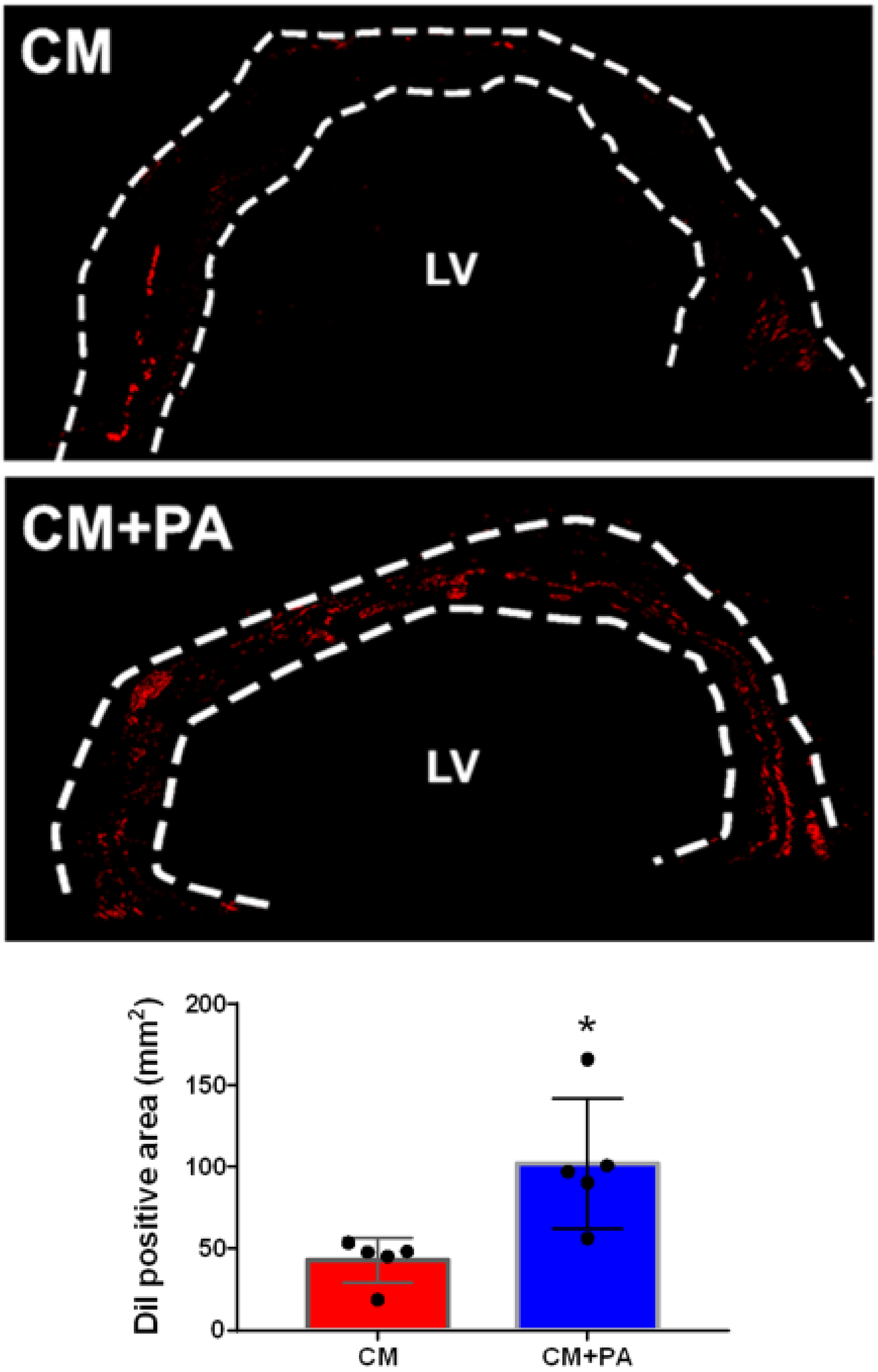

Supplementary Figure 10. Representative images of engrafted hiPSC-CMs (red) in the absence or presence of hMSC-PAs in MI heart. For tracing purpose, hiPSC-CMs were pre-labeled with Dil-CM (red) prior to injection to the MI hearts. Data are represented as mean ± SEM. **p* < 0.05 compared to CM group. n=10 biologically independent samples per group. T test was used for statistical analyses. CM: hiPSC-CMs, CM + PA: hiPSC-CMs + hMSC-loaded patch

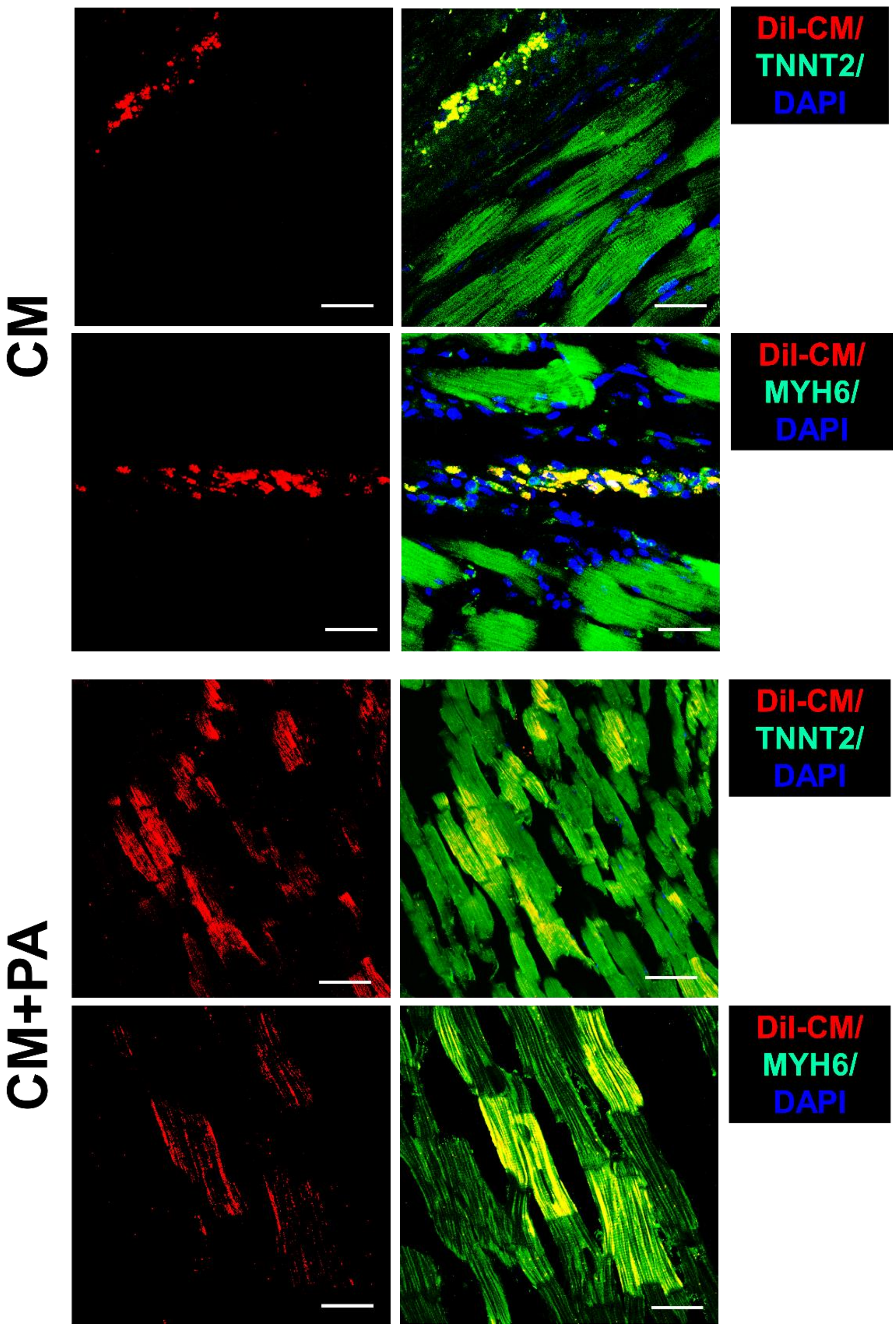

Supplementary Figure 11. Representative images of engrafted hiPSC-CMs in the absence or presence of hMSC-PAs in MI heart. For tracing purpose, hiPSC-CMs were pre-labeled with DiI-CM (red) prior to injection to the MI hearts. DiI-labeled hiPSC-CMs (red) expressed cardiac specific proteins including TNNT2 (green) and MYH6 (green) when they were injected in the absence or presence of hMSC-PAs in MI hearts. Scale bars: 10μm. CM: hiPSC-CMs, CM + PA: hiPSC-CMs + hMSC-loaded patch

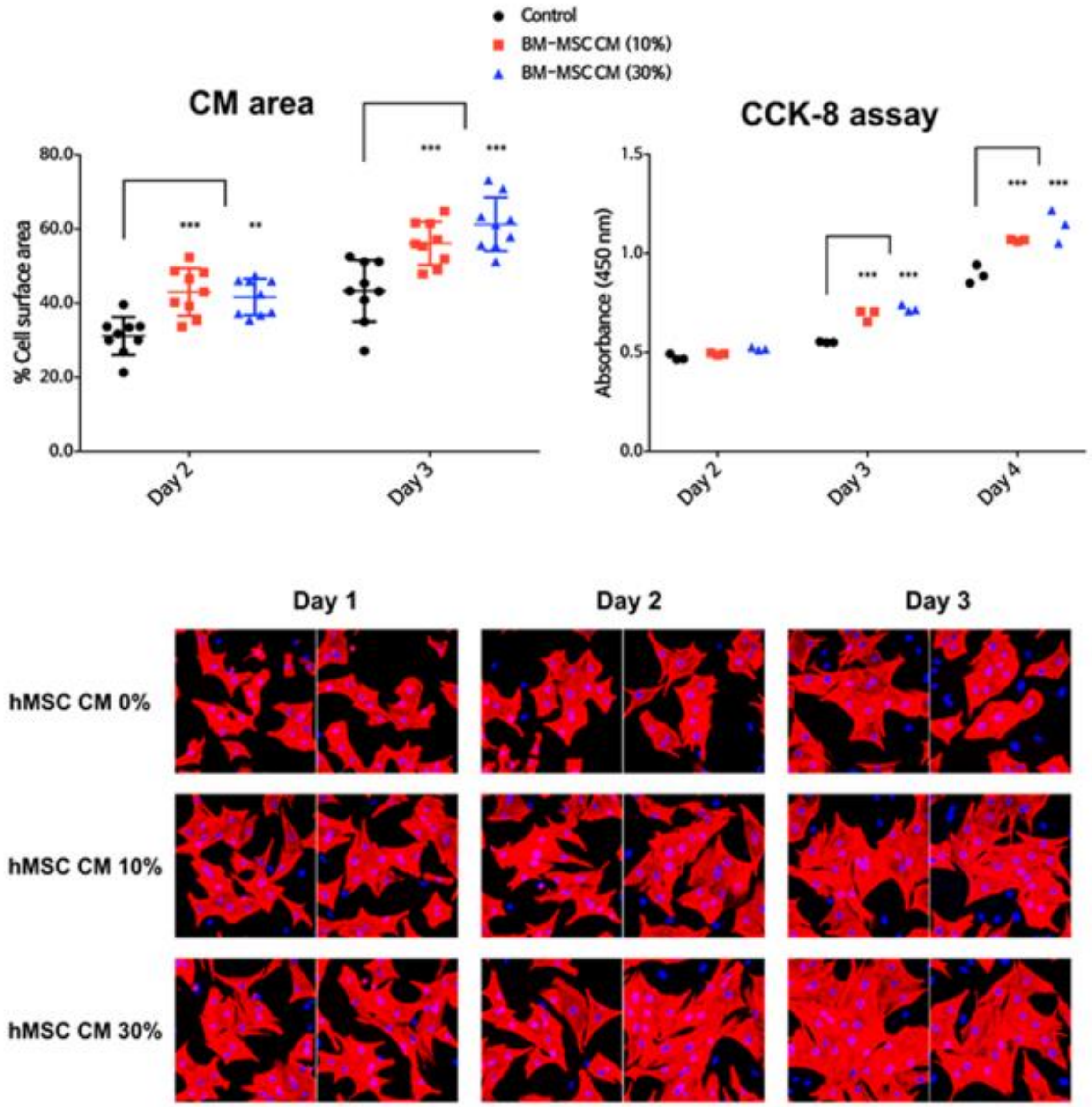

Supplementary Figure 12. Effects of hMSCs on cardiomyocyte maturation. Treatment with hMSC-conditioned media (hMSC-CA) collected from the hMSC cultures to the cultured neonatal rat ventricular cardiomyocytes (NRVM) significantly increased the size of NRVM determine by cardiomyocyte area measurement and the Cell Counting Kit-8 (CCK-8) array. Representative images of enlarged NRVM by treatment with hMSC CM. Data are represented as mean ± SEM. **p < 0.05 compared to control group, ***p < 0.01 compared to control group. n=3 biologically independent samples per group. One way ANOVA was used for statistical analyses.

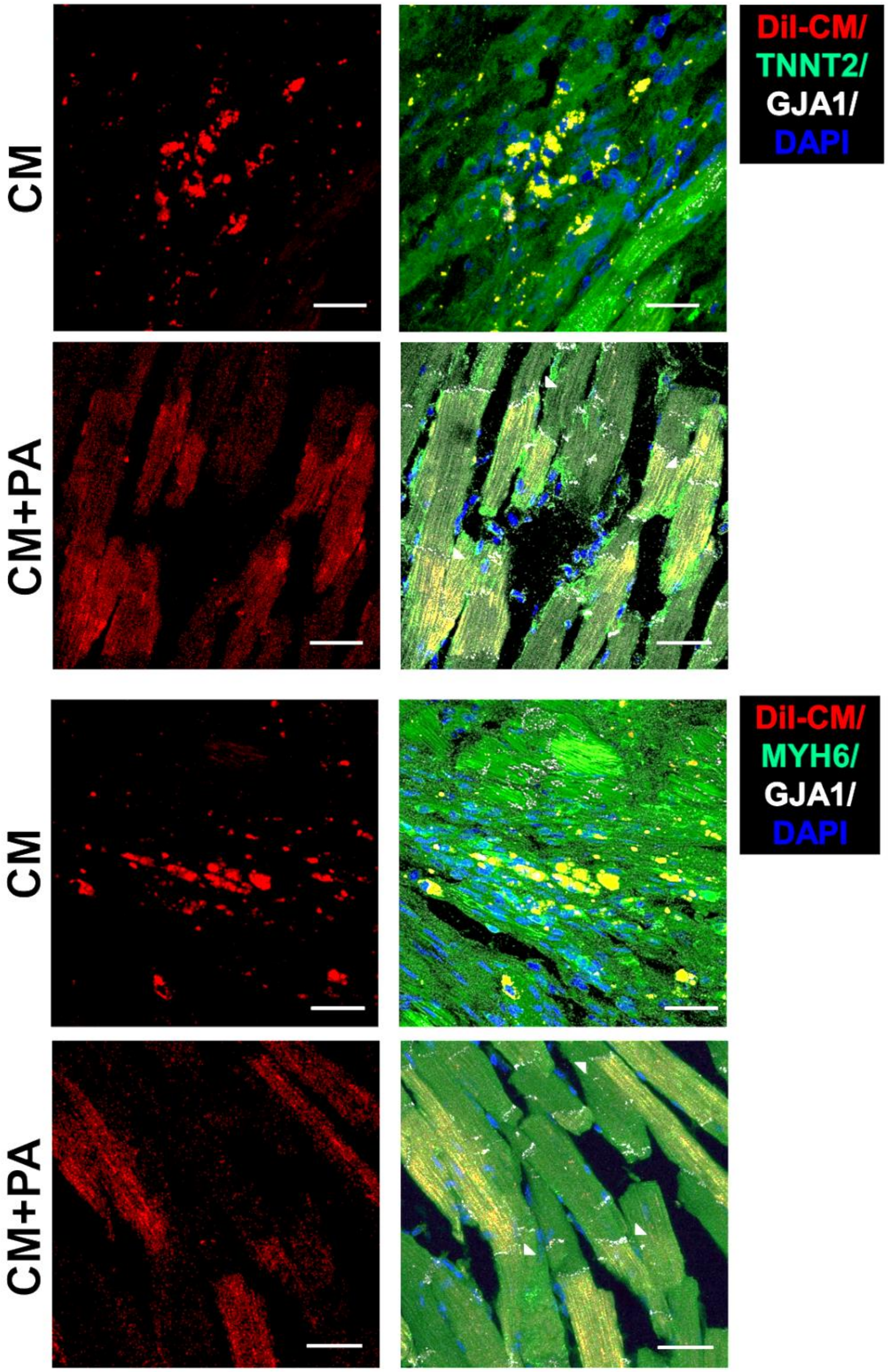

Supplementary figure 13. Implantation of hMSC-PAs improves the maturation of hiPSC-CMs on the infarcted myocardium. (A) DiI-labeled hiPSC-CMs (red) expressed cardiac specific proteins such as TNNT2 (green), MYH6 (green) and GJA1 (white) when they were injected in the absence or presence of hMSC-PAs in MI hearts. Expression of GJA1 indicates integration of implanted hiPSC-CMs with host myocardium. Scale bars: 10μm.

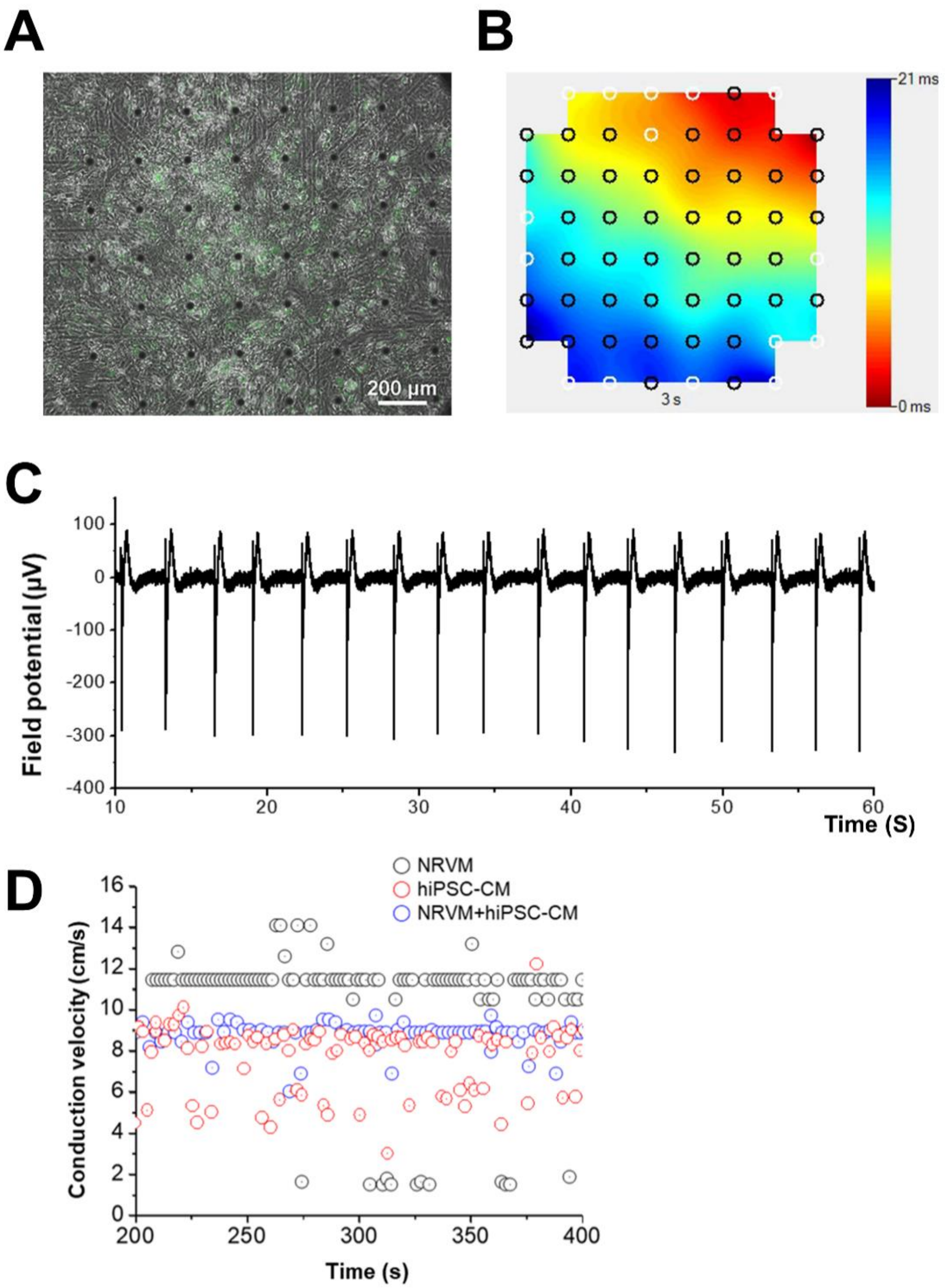

Supplementary figure 14. Analysis of electrical activity between hiPSC-CMs and neonatal rat ventricular cardiomyocytes through multielectrode arrays. (A) Field image of co-cultured neonatal rat ventricular cardiomyocytes (NRVM) and hiPSC-CMs on MEA chip. **(B)** Isochronal maps of spontaneous AP propagation recorded from co-cultured NRVM and hiPSC-CM for 200 s. The activation time is represented at the right of the map. **(C)** A representative MEA extracellular recording from co-cultured NRVM/hiPSC-CM. **(D)** Temporal development of conduction velocity from NRVM (black circle), hiPSC-CMs (red circle), and NRVM + hiPSC-CMs (blue circle)

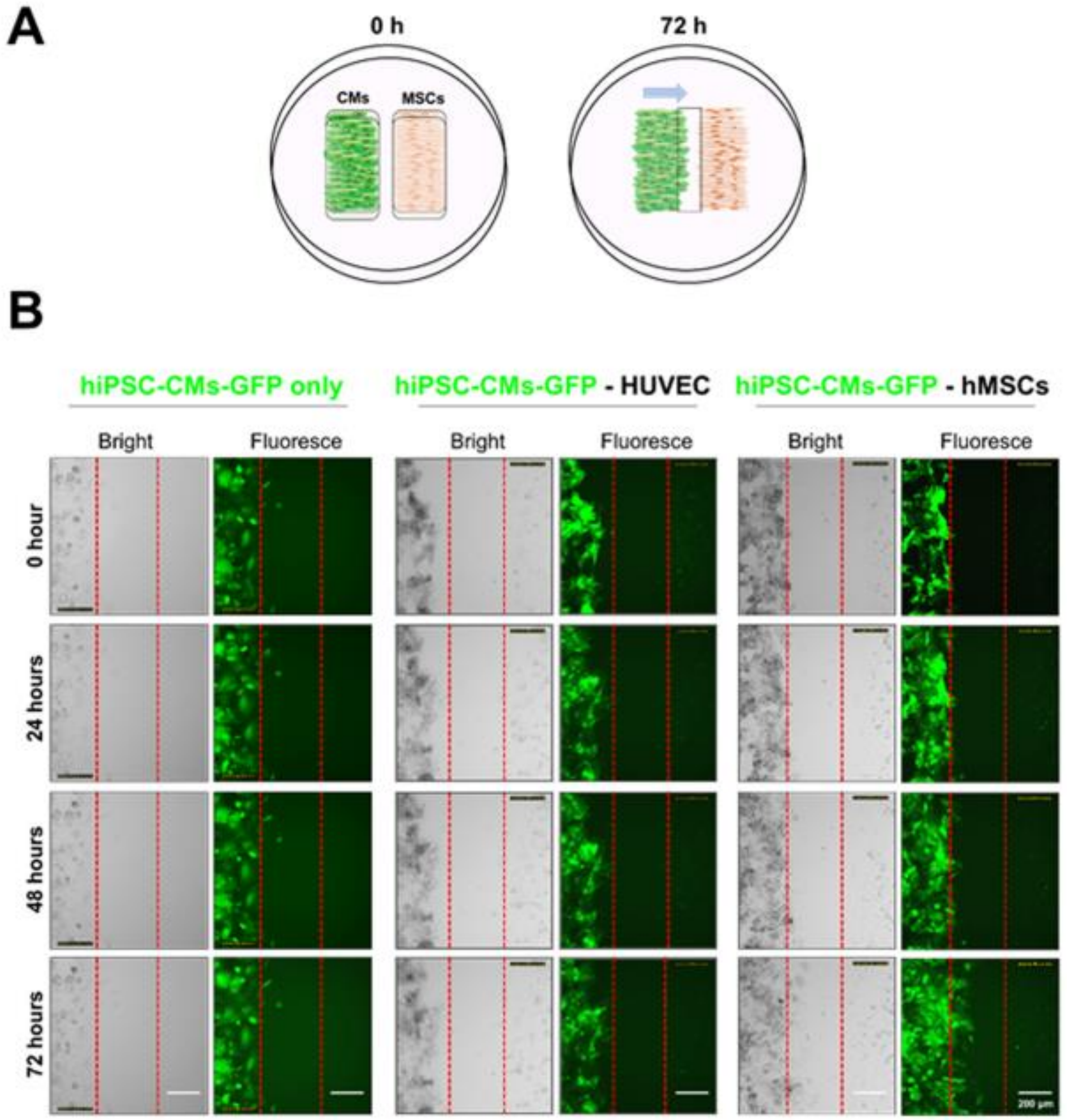

Supplementary Figure 15. Migration of hiPSC-CMs when they were co-cultured with hMSCs. (A) Schematic representation of monolayer co-culture system to assess the migration of hiPSC-CM to the hMSCs. **(B)** Representative images of hiPSC-CM-GFP culture alone or co-cultures of hiPSC-CM-GFP with HUVEC or hMSCs. Red box and white arrows indicate the migration area at specific time points and the hiPSC-CM-GFP. Scale bar: 200μm

Supplementary table 1. Primer sequences used for qRT-PCR Analysis

Target gene	Sequences	
	Forward (5'-3')	Reverse (5'-3')
18S rRNA	CGC GGT TCT ATT TTG TTG GT	AGT CGG CAT CGT TTA TGG TC
PLGF	CAG CCA ACA TCA CTA TGC AG	TCC TCT GAG TGG CTG GTT A
CD31	CTG GGA GGT ATC GAA TGG GC	CCC GAG ACT GAG GAA TGA CG
VEGFa	TTT CTC CGC TCT GAA CAA GGC	TGC AGA TCA TGC GGA TCA AAC
IGF-1	TGG TGG ACG CTC TTC AGT TC	AGT GTA CTT CCT TCT GAG TCT TGG
FGF-2	GAT CCC AAG CGG CTC TAC TG	TAG TTT GAC GTG TGG GTC GC
Ang1	CAC CGT GAG GAT GGA AGC CTA	TTC CCA AGC CAA TAT TCA CCA GA
Ang2	CAT GAT GTC ATC GCC CGA CT	TCC ATG TCA CAG TAG GCC TTG
Col I	GTA CAT CAG CCC AAA CCC CA	TCG CTT CCA TAC TCG AAC TGG
Col III	AGT GGC CAT AAT GGG GAA CG	CAG GGT TTC CAT CCC TTC CG
MMP2	GGG TGG TGG TCA CAG CTA TT	CCC AGC CAG TCC GAT TTG AT
MMP9	GAT CCC CAG AGC GTT ACT CG	GTT GTG GAA ACT CAC ACG CC
TIMP2	ATG GCA ACC CCA TCA AGA GG	CCG CCT TCC CTG CAA TTA GA
IL 1β	AGA AGA GCC CGT CCT CTG TGA	TCA GAC AGC ACG AGG CAT TT
IFNG	TGT CAT CGA ATC GCA CCT GA	TGT GGG TTG TTC ACC TCG AA
TNFα	GCA TGA TCC GAG ATG TGG AA	CAG ACA CCG CCT GGA GTT CT
IL10	GAA TTC CCT GGG AGA GAA GC	CGG GTG GTT CAA TTT TTC AT
TGFβ1	GCA ACA ATT CCT GGC GTT ACC	TTC CGT CTC CTT GGT TCA GC
