# Supplementary material for: Dual stem cell therapy synergistically improves cardiac function and vascular regeneration following myocardial infarction
Source: Nat Commun. 2019 Jul 16;10:3123. doi: 10.1038/s41467-019-11091-2 (PMC6635499; doi:10.1038/s41467-019-11091-2)
Supplement: Supplementary file 3 — Description of Additional Supplementary Files [file 41467_2019_11091_MOESM3_ESM.pdf]

Description of Additional Supplementary Files

Supplementary Movie 1. Contraction of hiPSC-derived cardiomyocytes 1

Supplementary Movie 2. Contraction of hiPSC-derived cardiomyocytes 2

Supplementary Movie 3. Surgical procedures

Supplementary Movie 4. Recording of echocardiography pre-intervention

Supplementary Movie 5. Recording of echocardiography 8 weeks post-intervention

Supplementary Movie 6. Contraction of hiPSC-CMs-GFP

Supplementary Movie 7. Recording of MEA mapping between hiPSC-CMs and NRVM

Supplementary Movie 8. Migration of hiPSC-CMs-GFP (hiPSC-CM-GFP culture Alone)

Supplementary Movie 9. Migration of hiPSC-CMs-GFP (co-cultures of hiPSC-CM-GFP with HUVEC)

Supplementary Movie 10. Migration of hiPSC-CMs-GFP (co-cultures of hiPSC-CM-GFP with hMSCs)
